# Facile Oil Removal from Water-in-Oil Stable Emulsions Using PU Foams

**DOI:** 10.3390/ma11122382

**Published:** 2018-11-27

**Authors:** Suset Barroso-Solares, Javier Pinto, Despina Fragouli, Athanassia Athanassiou

**Affiliations:** 1 Smart Materials, Istituto Italiano di Tecnologia, via Morego 30, 16163 Genova, Italy; jpinto@fmc.uva.es (J.P.); despina.fragouli@iit.it (D.F.); 2 Cellular Materials (CellMat) Research Group, Condensed Matter Physics Department, University of Valladolid, Paseo de Belen 7, 47011 Valladolid, Spain

**Keywords:** wetting properties, absorption, oil-water emulsions, polymer foams

## Abstract

Superhydrophobic and oleophilic polyurethane foams were obtained by spray-coating their surfaces with solutions of thermoplastic polyurethane and hydrophobic silicon oxide nanoparticles. The developed functionalized foams were exploited as reusable oil absorbents from stable water-in-oil emulsions. These foams were able to remove oil efficiently from a wide range of emulsions with oil contents from 10 to 80 v.%, stabilized using Span80. The modified foams could reach oil absorption capacities up to 29 g/g, becoming a suitable candidate for water-in-oil stable emulsions separation.

## 1. Introduction

Polymer foams are biphasic materials composed of a polymer matrix and a gaseous phase. However, this apparently simple definition hinders a vast complexity, in which the characteristics of each phase, the geometrical configuration of the phases (in terms of sizes or distribution), and the interactions between them play a decisive role in their physical properties [1]. The understanding of some of the underlying relationships of this complexity, achieved after decades of research, has proved polymer foams as highly versatile materials which are currently widely employed in transportation, thermal and acoustic insulation, packaging, commodity, or construction [1]. Nevertheless, further comprehension and exploitation of the polymer foams features can still expand their potential applications. On the one hand, recent works on the development of nanocellular polymers [2] and nanocomposite foams [3] enhanced the understanding of these materials and suggested new potential applications (e.g., transparent foams, high-performance thermal insulators, electromagnetic shielding, energy storage) [2,3,4,5]. On the other hand, a new generation of functional polymer foams have been proposed in the last years for several water remediation applications, a research field that has become a global priority (e.g., contaminated drinking water is estimated to cause half a million deaths each year) [6]. Polymer foams have been successfully proposed to remove heavy metal ions from water [7,8], as well as to develop bactericide water filters by introducing silver or copper nanoparticles [9,10,11,12] and have attracted a large interest and shown a great performance in water–oil separation applications [13]. 

Water–oil separation has become a worldwide need in numerous industrial production areas, as huge amounts of oily wastewaters are produced from diverse industrial process, presenting severe environmental and health risks [14]. Efficient separation approaches have been proposed by the development of simultaneously superhydrophobic and superoleophilic polymeric foams. These materials can exclusively absorb oil and repel water, presenting also high oil absorption capacities [13,15,16,17,18,19]. The majority of the proposed approaches have been focused on the absorption of free oil from water, simulating oil spills [13,15,16,17,18,19]. A large number of polymer foams have been employed as an in situ oil spills remediation approach, using them mainly as oil absorbents. These materials can be placed in the surface of the polluted water, where they absorb only the oil, and then recovered [13]. Although different polymer matrices have been tested with this aim, polyurethane and melamine foams have shown the best performances [13], as the very high porosity achievable by both kinds of foams led to very high oil absorption capacities [13,20]. Moreover, other parameters, such as the cell size and the cell connectivity, also play a significant role in the water–oil separation process [20]; while the improvement of the separation efficiency of these materials has been widely attempted by functionalization procedures [13].

However, oil spills and industrial oily wastewaters commonly do not present only free oil in the surface of the water but also emulsified oil (i.e., small oil droplets dispersed in water, or small water droplets dispersed in oil) [14]. This circumstance has been usually not taken into account by previous works using polymer foams in the remediation of oil spills, and the results of the free oil removal performance of these materials cannot be extrapolated to actual scenarios with emulsified oil. It is well-known that the separation of emulsions is more challenging than the separation of free oil from water, especially in the presence of surfactants that make the emulsions very stable [21,22]. Up to the knowledge of the authors, only a few recent works have studied the suitability of such polymeric foams for the separation of non-stabilized emulsions [23,24]. In particular, Wang and Zheng [23] have modified polyurethane (PU) foams with stearic acid by dip-coating in order to fabricate superhydrophobic/superoleophilic foams exhibiting excellent durability, high free oil absorption capacities up to 41.6 g/g, and also an acceptable surfactant-free oil-in-water emulsions separation efficiency (transparency of filtrate: >80%). Also, Wang et al. [24] proposed carbon nanotube/poly(dimethylsiloxane)-coated PU foams for the continuous separation of surfactant-free water-in-oil emulsions with high efficiency (recovered oil purity: >99.97 wt.%), demonstrating that, in non-stabilized emulsions, properly functionalized polymeric foams can be efficiently employed on gravity-driven separation processes [25,26]. However, the separation of stable water–oil emulsions employing porous absorbents has been scarcely reported in the literature. On the one hand, a few works employing other porous materials (e.g., fibrous mats, porous membranes) as oil absorbents report successful results when employed in stable water–oil emulsions [27,28,29,30,31]. On the other hand, just Li et al. [22] have proposed the use of polymer foams for the separation of surfactant-stabilized oil-in-water emulsions. They followed a multiple-step procedure to modify attapulgite particles, providing them a superhydrophobic/superhydrophilic behavior, and then transfer the particles to polyurethane foams. These functionalized polyurethane foams were tested in oil-in-water emulsions with a 50% volume percentage of oil and stabilized using Tween 80. The separation procedure was carried out by compressing and agitating the PU foams in the emulsions, reaching separation efficiency about 99% in that particular conditions. 

It has been widely proved that polymer foams are suitable candidates for the separation of free oil and non-stabilized emulsions. However, there are just a few evidences about their use on stable emulsions, which are very common in actual remediation scenarios (e.g., some components of crude oils are known to stabilize emulsions in oil spills). Thus, it still should be proved if polymer foams, preferably obtained by facile functionalization procedures, could be employed in the separation of stable emulsions with a wide range of oil contents. Moreover, the incorporation of fillers or coatings to the polymer foams could also modify their mechanical properties [32,33], being necessary to ensure that the functionalization procedure does not damage the mechanical performance of the foams. 

Herein, we proposed the use of functionalized polymer foams for the separation of stable water-in-oil emulsions presenting a wide range of oil contents. We obtained simultaneously superhydrophobic and oleophilic polyurethane foams via a facile single-step route, by spray-coating their surfaces with a homogeneous solution of thermoplastic polyurethane (TPU) and hydrophobic silicon oxide nanoparticles (SNPs) in chloroform. The developed functional foams presented high oil separation efficiency in stable water-in-oil emulsions, while their mechanical properties were not affected by the functionalization procedure. Moreover, these foams could be reused for at least 50 cycles of oil absorption/oil recovery achieving a stable performance, proving that the developed systems can be used as efficient absorbents in wastewater and oil-polluted water treatment even if stable emulsions are present.

## 2. Materials and Methods

### 2.1. Materials

Polyurethane foams with the same porosity (0.975) and different pore sizes (Φ) (see Table 1), provided by Recticel Flexible Foams Inc (Wetteren, Belgium), were used in this study. Thermoplastic polyurethane (TPU, Elastollan^®^ 1185A, BASF) was employed as coating of the foams’ surfaces. Hydrophobic silicon oxide fumed nanoparticles (SNPs, AEROSIL^®^ R 812, ρ ≈ 2 g/cm^3^ at 20 °C, Evonik Industries AG) with sizes between 5 and 40 nm were employed as nanofillers in the TPU coating. Mineral oil (ρ = 0.84 g/cm^3^ a 25 °C, Sigma–Aldrich) and distilled water were mixed to obtain emulsions, using Span80 (Sorbitan monooleate, non-ionic, viscosity 1200–2000 mPa∙s at 20 °C, Hydrophile-Lipophile Balance (HLB) value 4.3 ± 1.0, ρ = 0.986 g/cm^3^ at 25 °C, Sigma–Aldrich) as water-in-oil emulsion stabilizer. Chloroform (CHCl_3_, Purity, Gas chromatography (GC) > 99.8%, ρ = 1.48 g/cm^3^ at 20 °C, Sigma-Aldrich) was used as received as common solvent for the TPU and SNPs, whereas ethanol (Ethyl alcohol-d6, Purity (GC) > 99.5%, ρ = 0.892 g/cm^3^ at 25 °C, Sigma-Aldrich) was employed for the rinsing of the treated foams.

### 2.2. Fabrication of Treated PU Foams (PUT)

The external surfaces of PU foam samples cut in cubes of 1 cm^3^ were coated using a spray-coating set-up [34]. Each one of the six facets of the foam samples was coated with a 3 mL solution of 2.5 mg/mL hydrophobic silicon oxide fumed nanoparticles (SNPs) and 7.5 mg/mL thermoplastic polyurethane in chloroform. The distance between the nozzle head and the samples were modified obtaining treated PU foams (PUT) with different surface roughness after drying at room temperature (RT). A distance of 20 cm was identified as optimal to carry out the procedure, providing a proper roughness increase and coating stability (see Supplementary Materials, Figure S1). All samples were allowed to dry at room temperature, and then washed by immersion in ethanol for 30 min under sonication operating at 59 kHz (Labsonic LBS2, FALC Instruments). Then, the samples were rinsed with water and mechanically squeezed, up to 90% of deformation, with the aim to remove the remaining water and any unattached coating (see Supplementary Materials, Figure S1). 

### 2.3. Preparation of Emulsions

Stable emulsions were prepared following the procedure described in a previous work, where the stability of these emulsions was also studied [28]. First, Span80 was dissolved into the oil phase (0.5 wt.%) by shaking for 24 h. Then, the aqueous phase was added to the mixture to form the Span80-stabilized emulsions and dispersed using a high-intensity ultrasonication tip (VCX 750, Vibra cell, SONICS) at 40% amplitude for 15 s at room temperature. The concentrations of the emulsions are expressed using the volume percentage of the oil (v.%). Six-mL water-in-oil emulsions were prepared for the absorption tests with oil contents of 10, 30, 50, and 80 v.%, which correspond to oil amounts of 0.504, 1.512, 2.520, and 4.032 g, respectively). All these emulsions are rather stable, presenting no changes after 30 min as proved elsewhere [28].

### 2.4. Characterization

The weight increase (*wt*.%) of the foams due to the functionalization procedure was determined by weighing the samples before (*w_PU_*) and after the treatment (*w_PUT_*) (Equation (1)).

(1) $$wt.\%=\frac{\left(w_{PUT}-w_{PU}\right)}{w_{PU}}\cdot 100,$$

The foams’ morphology was examined by scanning electron microscopy (SEM, JEOL Model JSM-6490) (Boston, MA, USA), being the samples previously coated with gold. Moreover, a KSVCAM200 (Kruss, Germany) contact angle goniometer was used to determine the apparent contact angle of the foams for water (AWCA) and oil (AOCA), averaging five measurements for each kind of sample, using 5-μl drops at RT. As the measurement of the contact angle in polymer foams can be affected by the pore size the obtained values are defined as “apparent” contact angles (see Supplementary Materials, Figure S4) [20]. 

Oil and water absorption capacity (respectively *C_oil_* and *C_water_*), as well as the simultaneous oil and water uptakes from emulsions (respectively *w_oil_* and *w_water_*), of both pristine and treated foams were determined with samples of 1 cm^3^ in contact with 6 mL of the tested liquid for 15 min.

The water and oil absorption capacity (*C_water_* and *C_oil_*) were obtained after immersing the foams in water or oil, while the simultaneous water and oil uptake (*w_water_* and *w_oil_*) were obtained after immersing the foams in water-in-oil emulsions [28]. All these values are expressed as the mass of the absorbed liquid divided by the mass of the absorbent (g/g). Due to the buoyancy of the samples, and to increase the contact between the foams and the emulsions, the foams were forced and kept below the surface of the water during the absorption experiments (See Supplementary Material, Figure S5).

On the one hand, C_water_ and C_oil_ were calculated using Equations (2) and (3), respectively.

(2) $$C_{water\;}\left[g/g\right]=\frac{w_{abswater}-w_{foam}}{w_{foam}},$$

(3) $$C_{oil}\;\left[g/g\right]=\frac{w_{absoil}-w_{foam}}{w_{foam}},$$

where *w*_foam_ is the initial weight of the foams, and *w*_abswater_ and *w*_foam_ are the weights of the foams after being in contact with the water or the oil, respectively.

On the other hand, the simultaneous water (*w*_water_) and oil (*w*_oil_) uptakes from emulsions were calculated using Equations (4) and (5), respectively.

(4) $$w_{water\;}\left[g/g\right]=\frac{Abs_{1}-Abs_{2}}{w_{foam}},$$

(5) $$w_{oil}\;\left[g/g\right]=\frac{Abs_{2}-w_{foam}}{w_{foam}},$$

where the foams’ weight were measured before (*w*_foam_) and immediately after being extracted from the emulsions (*Abs_1_*). Then, the samples were stored in an oven at 50 °C for one week, with the aim to ensure the complete water evaporation from the samples and weighted again (*Abs_2_*). Following this procedure, it was possible to identify separately the amount of absorbed oil (*w_oil_*) and water (*w_water_*).

The optimal contact time of 15 min between the foams and the emulsions was established by analyzing the evolution of the water and oil uptakes with the contact time. It was found that the best oil absorption selectivity was achieved after 15 min (i.e., simultaneous higher oil uptake and lower water uptake). Shorter contact times led to incomplete foam saturation, while longer times decreased the oil absorption selectivity (i.e., the oil uptake decreased, and the water uptake increased). Accordingly, this contact time was employed for all the oil absorption tests from emulsions, as well as for the reference oil and water absorption capacity tests.

The absorption selectivity of the foams towards the oil (%) was determined by analyzing the composition of the liquid absorbed by the foams from the emulsions. Accordingly, the absorption selectivity (%) was defined as the percentage of oil uptake (w_oil_) present on the liquid absorbed by the foam (*w*_oil_ + *w*_water_), as shown in Equation (6).

(6) $$Absorption\;selectivity\;\%=\frac{w_{oil}}{w_{oil}+w_{water}}\cdot 100,$$

Finally, it was studied the potential reusability of the PUT foams. Their oil absorption efficiency was analyzed for 50 absorption cycles for the emulsions presenting the higher and lower oil contents and 5 absorption cycles for the intermediate emulsions, being the samples rinsed with ethanol and dried subsequently to each absorption cycle.

Additionally, the functionalization stability was studied for 50 washing/drying cycles with ethanol by Fourier-transform infrared (FTIR) spectroscopy using a Bruker Tensor 27 spectrometer equipped with a MKII Golden-Gate diamond attenuated total reflectance (ATR) unit at room temperature in the range of 600–4000 cm^−1^. Also, the mechanical properties of the pristine (PU) and treated foams (PUT) were evaluated using samples with dimensions of 1 × 1 × 1 cm^3^ through cyclic compressive tests at RT using a PerkinElmer DMA7 dynamic mechanical analyzer (DMA) with a parallel-plate system (top plate of 12 mm in diameter). Samples were tested in force control at 20 °C, applying an increasing force (500 mN/min) up to 2 N and then allowing the recovery of the sample without load for two minutes. Test were performed for a duration of 33 cycles. 

## 3. Results and Discussion

First, it was identified the optimal porous structure among the different PU foams (see Table 1) to be favorable towards oil absorption and unfavorable towards water absorption. Thus, the absorption of the pristine foams was separately tested in water or oil. It was found that the highest *C_oil_* (~30.5 g/g) and the lowest *C_water_* (~15.6 g/g) were obtained for the PU foams with the smallest average pore size (435 µm, see Table 1), in good agreement with previous works [13]. Additionally, all the foams presented a superoleophilic behavior (AOCA ≈ 0°), whereas an increase of the AWCA was measured by decreasing the pore size (Table 1), raising from 98° to 133° when the pore size of the PU foams was modified from 1741 µm to 435 µm.

**Figure 1:**
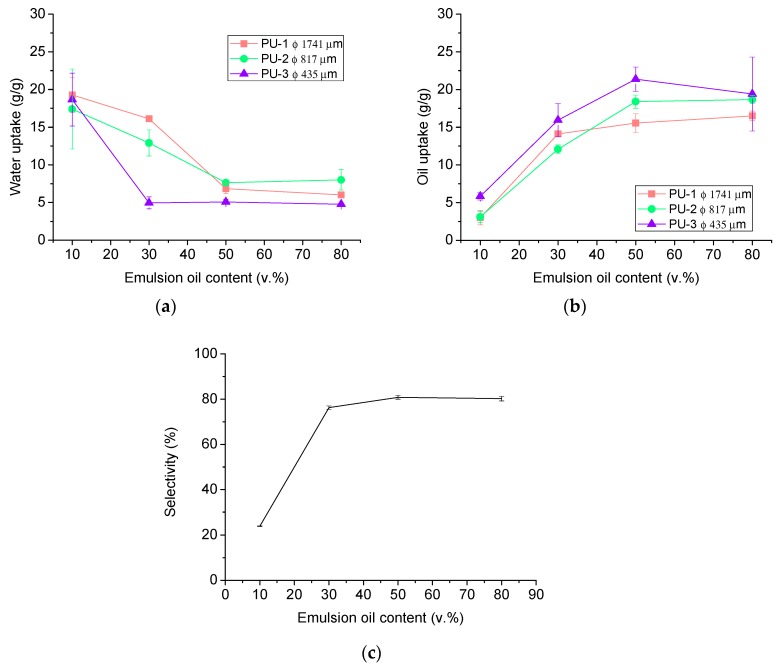
Simultaneous (**a**) water (w_water_) and (**b**) oil uptake (w_oil_) from stable water-in-oil emulsions using pristine PU foams with different pore sizes. Oil absorption selectivity (**c**) of PU-3 (Φ = 435 µm) from stable water-in-oil emulsions.

This trend was further verified by testing the pristine foams with stable water-in-oil emulsions presenting oil contents ranging from 10 to 80 v.%. It was found that for all the PU foam under study the evolution of the simultaneous water (*w_water_*) and oil uptakes (*w_oil_*) was clearly related to the composition of the water-in-oil emulsion employed. After being in contact with emulsions with low oil contents, the pristine PU foams absorbed high amounts of water and small amounts of oil; while high oil contents led into higher oil than water absorption. Regarding the influence of the pore size, the foams with the smallest pore size obtained the lowest *w_water_* simultaneously with the greatest *w_oil_*, regardless of the emulsion tested (Figure 1a,b), confirming that the reduction of the pore size was also improving the performance of the foams when tested in stable emulsions. However, even for the smallest pore size, the selectivity towards oil absorption was not optimal (i.e., a significant amount of water was also absorbed, particularly from emulsions with low oil content (10 v.%)) (Figure 1c). For this reason, it was necessary to functionalize the surface of the foams aiming to improve their selectivity.

**Figure 2:**
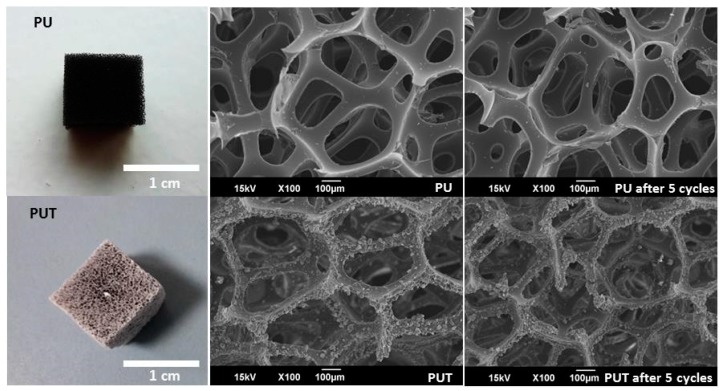
Photographs (**left**) of PU (**up**) and treated PU foams (PUT) (**down**) foams, and SEM micrographs of their porous structure before (**middle**) and after five washing cycles with ethanol (**right**).

Therefore, samples of the PU-3 foam, with the smallest pore size, were functionalized by spray-coating with TPU and SNPs starting from chloroform solutions (see Materials and Methods). This treatment did not modify the porosity of the foams or the pore size (Figure 2), but it slightly increased the mass of the foams by 4.54 ± 1.11 wt.% and the roughness of the pores’ walls and the external surfaces of the foams. These modifications were stable even after five washing cycles of the foams with ethanol (Figure 2).

The modification of the external surfaces of the foams increased the AWCA by 15° (from 133.3 ± 4.4° (PU-3) to 149.9 ± 1.8° (PUT)) while maintaining the superoleophilicity. The applied coating was stable and provided a constant AWCA after five washing cycles with ethanol. This change in the wettability of the modified foams was responsible for the drastic reduction of their water absorption, achieving almost negligible *C_water_* (0.20 g/g) compared to the untreated foams (15.64 g/g) when the samples were tested in water. At the same time, the new *C_oil_* (29.08 g/g) remained almost unaltered compared to the untreated foams (30.50 g/g) after being placed on the surface of the oil, being the slight decrease related to the total weight increase of the foams after the treatment (see Supporting Information, Figure S3).

**Figure 3:**
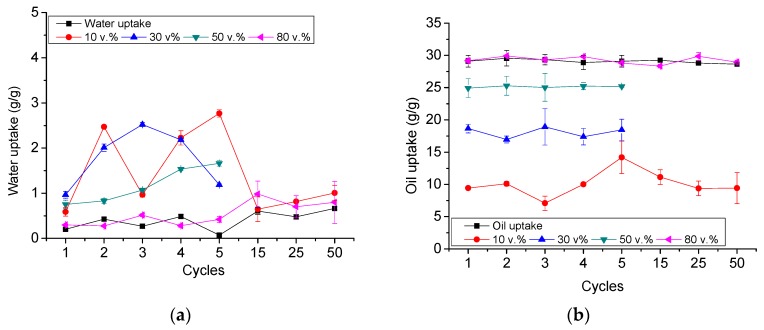
Simultaneous (**a**) water (w_water_) and (**b**) oil uptakes (w_oil_) of PUT foams from stable water-in-oil emulsions up to 50 absorption/washing cycles.

An improved and stable performance, compared to the untreated PU foams, was also found when the PUTs were used in stabilized water-in-oil emulsions. This improved behavior was confirmed for up to 50 immersion-ethanol washing cycles (Figure 3). It was found that the *w_water_* decreased for all the emulsions, reaching values below 2.5 g/g, independently of the emulsion (Figure 3a), well below the values obtained without treatment (from 5.0 to 20.0 g/g, see Figure 1a). Furthermore, the *w_oil_* increased for all the emulsions, reaching values near or over 20.0 g/g for emulsions with oil contents over 30 v.% (Figure 3b). It should be noticed that the amount of oil present in the emulsions with 10 wt.% oil (0.504 g) was significantly lower than the amount of oil that the foams employed in the tests could absorb (about 0.8–1.0 g), and therefore, the maximum *w_oil_* that could be reached in this case was just ~15 g/g.

**Figure 4:**
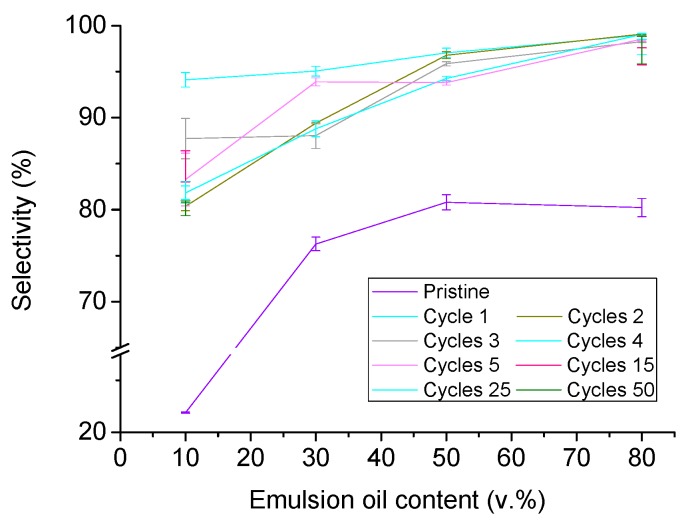
Oil absorption selectivity of PUT foams from stable water-in-oil emulsions up to 50 absorption/washing cycles.

Also, the analysis of the absorption selectivity of the foams towards the oil provides clear evidence of the beneficies of the functionalization procedure. The pristine PU-3 foams (see Figure 1c) did not present a proper absorption selectivity, being their selectivity at low oil contests about only 20%, reaching maximum selectivity values of about 75–80% at medium–high oil contents. On the contrary, PUT foams presented a clear selective absorption behavior independently of the oil content (see Figure 4). It was found that for the first use, the selectivity towards oil absorption of the PUT was higher than 95%, independently of the starting emulsion (even in emulsions with only 10 v.% of oil) (Figure 4). In the following cycles, only a slight decrease in the selectivity was found, eventually obtaining a stable selectivity up to 50 cycles over 80% for emulsions with 10 and 30 v.% oil content, and over 90% for emulsions with 50 and 80 v.% oil content.

**Figure 5:**
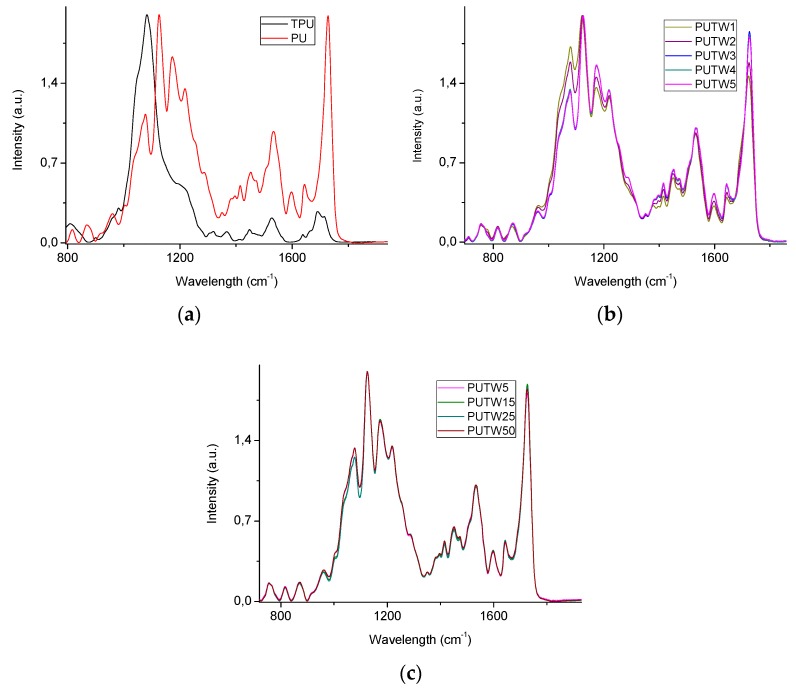
FTIR spectra of (**a**) PU foams and thermoplastic polyurethane (TPU), (**b**) PUT samples after 1 (PUTW1), 2 (PUTW2), 3 (PUTW3), 4 (PUTW4), and 5 (PUTW5) washing cycles), and (**c**) PUT samples after 5, 15 (PUTW15), 25 (PUTW20), and 50 (PUTW50) washing cycles. Full spectra can be found in the Supplementary Materials (see Figure S7).

Further work was carried out in order to characterize the stability of the treatment of the PUT foams. First, an FITR study was carried out with PUT samples subjected up to 50 washing cycles with ethanol. It was analyzed the evolution of the TPU presence on the surface of the PUT foams as an indicator of the presence of the treatment on these surfaces. The ratio between the PUT foams peaks respectively at about 1078 cm^−1^ (*I_1078_*, contributed by both the PU and TPU, see Figure 5a) and 1124 cm^−1^ (*I_1124_*, contributed mainly by the PU, see Figure 5a), both peaks related to vibrations of C–O–C groups [35,36], was employed with this aim.

It was found that there was a slight treatment lost from the first to the third washing cycles (Figure 5b), as the *I_1078_*/*I_1124_* ratio decreased from 0.86 to 0.66. This result is in good agreement with the oil absorption selectivity of the samples, which also showed a slight decrease after the first absorption/washing cycles (see Figure 4) but then reached a rather stable performance in the following cycles. Moreover, this study was extended up to 50 washing cycles, finding that the *I_1078_*/*I_1124_* ratio was rather constant after the first cycles, presenting values between 0.62–0.66. Although this slight loss of the coating presents an effect on the selectivity of the first cycles, it was found to be no significant in terms of the weight of the foam (see Supplementary Materials, Table S1). Moreover, no nanoparticles release was found during these tests (see Supplementary Materials, Table S1). Therefore, it was demonstrated that the functionalization procedure followed to produce the PUT foams was rather stable, at least up to 50 washing cycles.

**Figure 6:**
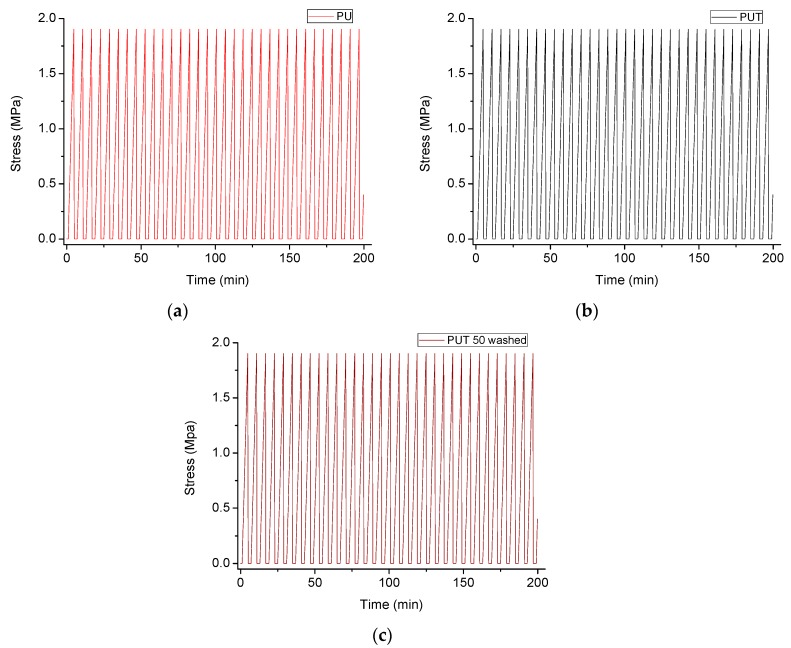
Stress–time curves for 33 deformation cycles of (**a**) pristine PU foams, (**b**) functionalized PUT foams, and (**c**) functionalized PUT foams after 50 washing cycles.

Finally, as the compression of polymer foams has been proposed in the literature as a suitable procedure to recover the absorbed oil [34], the mechanical behavior of the PU foams before and after the treatment was analyzed. In particular, pristine PU foams, functionalized PUT foams, and functionalized PUT foams after 50 washing cycles were subjected to 33 successive compression cycles (see Materials and Methods). As Figure 6 clearly shows, no clear difference in the stress relaxation due to viscous deformation after 33 compressive deformation cycles was found between any of the studied samples. Therefore, it was proved that neither the treatment procedure, nor the washing procedure modified the mechanical behavior of the employed PU foams, being the obtained PUT foams mechanically and chemically stable.

## 4. Conclusions

Functionalized PU foams with optimized wetting properties and oil separation efficiency from stable water-in-oil emulsions were obtained by a single-step spray-coating, using silica nanoparticles and thermoplastic polyurethane as a nanocomposite coating. These foams reached a maximum oil absorption capacity of about 30 g/g, as well as a selectivity above 95% for stable emulsions with oil contents from 10 to 80 v.%. Also, the stability of the coating and the mechanical properties of the foam, proved to be stable up to 50 washing cycles, allowed the reuse of the treated foams after ethanol washing up to at least 50 absorption cycles without a significant performance loss, making these functional foams efficient oil absorbents for oily wastewaters, including emulsions stabilized by a surfactant.

## Figures and Tables

**Table 1:** Polyurethane (PU) pristine foams’ properties: pore size, apparent water contact angle (AWCA), and water and oil absorption capacities.

Foam	Pore Size (µm)	AWCA (°)	*C_water_* (g/g)	*C_oil_* (g/g)
**PU-1**	1741	98.4 ± 11.0	28.94 ± 0.14	23.25 ± 0.10
**PU-2**	817	122.8 ± 7.6	31.01 ± 2.95	29.62 ± 0.24
**PU-3**	435	133.3 ± 4.4	15.64 ± 3.18	30.50 ± 0.01

## Supplementary Materials

The following are available online at http://www.mdpi.com/1996-1944/11/12/2382/s1. **Figure S1**. Spray-coating set-up. **Figure S2** Optical micrographs of PUT obtained by modifying the distance between the nozzle head and the samples. **Figure S3**. UV-Vis spectra of silica nanoparticles dispersed in ethanol (black), remaining ethanol after being in contact with treated foams not subjected to the initial washing procedure (red), and remaining ethanol after being in contact with treated foams subjected to the initial washing procedure and absorption tests (blue). **Figure S4**. Water drop placed in a PU pore, where θ is the real contact angle of water (WCA), and γ is the apparent contact angle (APCA) measured by the optical contact angle goniometer (a). Figures (b) and (c) illustrate the influence of the pore size on the apparent contact angle of water drops with the same size. **Figure S5**. Schematic representation (a) and photograph (b) of the absorption tests set-up. **Figure S6.** Water and oil absorption capacity using PU and PUT foams. **Figure S7.** FTIR spectra of PU foams and TPU (a), PUT samples after 1 (PUTW1), 2 (PUTW2), 3(PUTW3), 4 (PUTW4), and 5 (PUTW5) washing cycles (b), and PUT samples after 5, 15 (PUTW15), 25(PUTW20), and 50 (PUTW50) washing cycles (c). **Table S1.** Mass Increase of PUT foams obtained using different spray-coating distances and their stability during five consecutive washing cycles. The first one is part of the production route of the treated foams, as described in section 2.2, while the following four cycles are perfomed to study the stability of the treatment once the treated foams has been produced. **Table S2.** Oil and water absorption capacity of PU and PUT foams.
