# Supplementary material for: Facile Oil Removal from Water-in-Oil Stable Emulsions Using PU Foams
Source: Materials (Basel). 2018 Nov 27;11(12):2382. doi: 10.3390/ma11122382 (PMC6316968; doi:10.3390/ma11122382)
Supplement: Supplementary file 1 [file materials-11-02382-s001.pdf]

materials

Supporting information

Facile Oil Removal from Water-in-Oil Stable Emulsions Using PU Foams

Suset Barroso-Solares[1,2]*, Javier Pinto[1,2], Despina Fragouli[1] and Athanassia Athanassiou[1]*

[1] Smart Materials, Istituto Italiano di Tecnologia, via Morego 30, 16163 Genova, Italy; despina.fragouli@iit.it
[2] Cellular Materials (CellMat) Research Group, Condensed Matter Physics Department, University of Valladolid, Paseo de Belen 7, 47011 Valladolid, Spain; jpinto@fmc.uva.es
* Correspondence: sbarroso@fmc.uva.es(S.B.S.); athanassia.athanassiou@iit.it(A.A.)

Received: 1 November 2018; Accepted: 26 November 2018; Published: date

S1. Functionalization PU foams.

The external surfaces of polyurethane (PU) foam samples of 1 cm^3 were coated using a spray-coating set-up[1] (Figure S1). The distance between the nozzle head and the samples were modified obtaining treated PU foams (PUT) with different surface roughness after drying at room temperature (RT) (Figure S2).

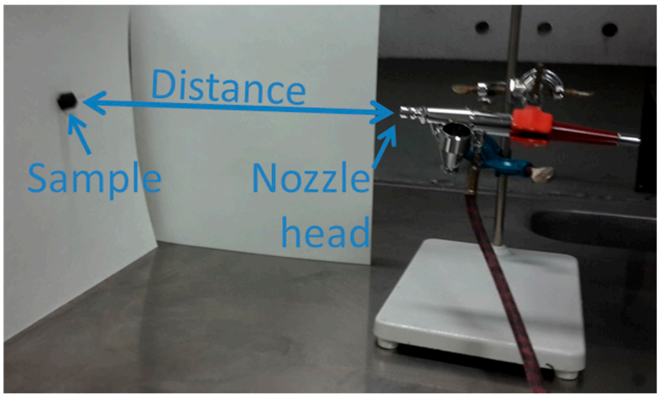

Figure S1. Spray-coating set-up.

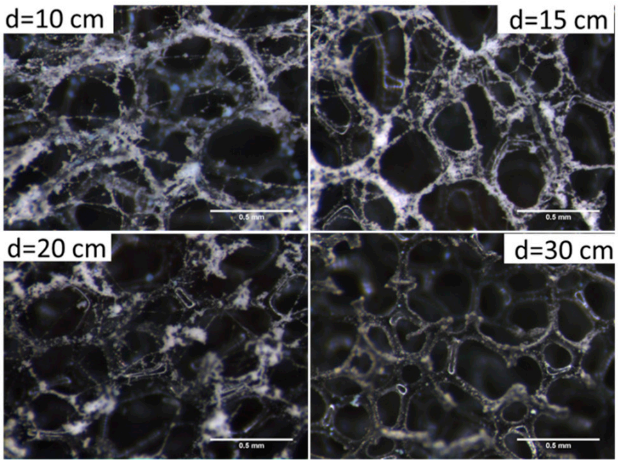

Figure S2 Optical micrographs of PUT obtained by modifying the distance between the nozzle head and the samples.

After drying the PUTs were washed by immersion in ethanol for 30 min under sonication operating at 59 kHz (Labsonic LBS2, FALC Instruments). Then, the samples were rinsed with water with water and mechanically squeezed, up to 90% of deformation, with the aim to remove the remaining water and any unattached coating. This procedure was carried out up to five times to check the stability of the treatment. The weight increase related to the treatment was studied after each washing cycle, using equation 1 (Table S1).

$$\boldsymbol{wt.\%} = \frac{(w_{PUT} - w_{PU})}{w_{PU}} * \mathbf{100}, \quad (1)$$

Table S1. Mass Increase of PUT foams obtained using different spray-coating distances and their stability during five consecutive washing cycles. The first one is part of the production route of the treated foams, as described in section 2.2, while the following four cycles are perfomed to study the stability of the treatment once the treated foams has been produced.

**Foams**	**Remaining coating (wt.%)**			
	**Treated foam after the dip-coating procedure**	**First washing cycle (part of fabrication procedure)**	**2 additional washing cycles**	**4 additional washing cycles**
Spraying using TPU and SNPs 10 cm	40.25 ± 5.01	18.38 ± 1.85	13.14 ± 1.28	13.12 ± 1.75
Spraying using TPU and SNPs 15 cm	30.25 ± 5.01	13.32 ± 1.59	9.64 ± 1.81	9.18 ± 2.22
Spraying using TPU and SNPs 20 cm	16.10 ± 1.25	4.54 ± 1.11	4.54 ± 1.11	4.53 ± 0.97
Spraying using TPU and SNPs 30 cm	10.25 ± 5.01	3.48 ± 1.15	3.07 ± 1.61	3.05 ± 0.52

As Table S1 shows, the first washing it is always required prior to further use, as a significant amount of the coating is released at this step. It should be noted that the complete characterization of the treated foams provided in this work was performed after the washing cycle included in the fabrication procedure, once the weight of the coating transferred to the foams is stable independently of the additional washing or absorption/washing cycles performed.

It was found that a spray-coating distance of 20 cm was optimal regarding the coating stability, being the coating completely stable during the successive washing cycles. On the one hand, coatings obtained with distances of 10 and 15 cm achieved higher amounts of remaining coating, but these coatings were not completely stable after the first washing cycles and presented higher weight losses than the coating obtained using a distance of 20 cm. On the other hand, coatings achieved employing distances of 30 cm were found to be also stable after the first washing cycle, but providing a lower amount of remaining coating. According to these results, and the appropriate wettability shown by these foams (see Results and Discussion section), this distance was selected to produce the PUT foams employed for oil absorption from water-in-oil stable emulsions.

In addition, the potential release of nanoparticles from the coating was studied during the washing cycles performed with ethanol after each absorption test. With this aim, the presence of

nanoparticles in the remaining ethanol after the washing of the foams was studied by UV-Vis spectroscopy, using a Varian CARY 300 Scan UV-visible spectrophotometer (Agilent Technologies, Santa Clara, CA, USA). This procedure was followed both with samples without and with the initial washing procedure with ethanol intended to remove the unattached particles transferred to the foam. Moreover, a dispersion of silica nanoparticles in ethanol was prepared and analyzed as a reference.

Figure S3 shows the obtained results. It was found that the samples not subjected to the initial washing procedure (described in the fabrication procedure of the treated foams and previously identified as necessary to remove unattached coating) present a significant release of nanoparticles. On the contrary, no nanoparticles release was found for treated foams after this initial washing, neither after the successive absorption/washing cycles.

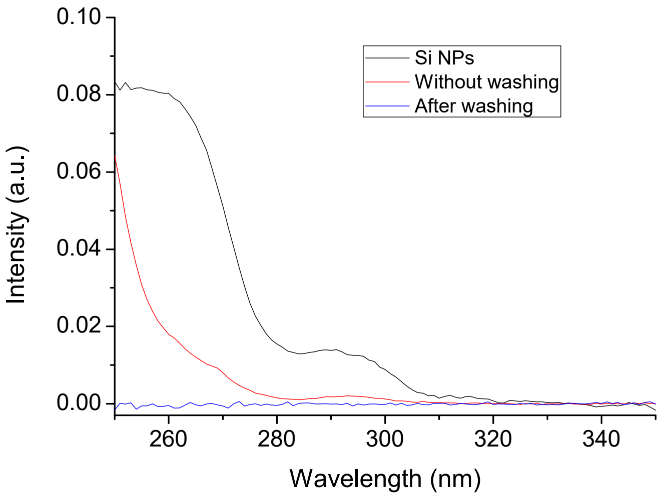

Figure S3. UV-Vis spectra of silica nanoparticles dispersed in ethanol (black), remaining ethanol after being in contact with treated foams not subjected to the initial washing procedure (red), and remaining ethanol after being in contact with treated foams subjected to the initial washing procedure and absorption tests (blue).

S2. Measurement of the contact angle in polymer foams.

Previous works demonstrated that measuring the contact angle in porous surfaces present some difficulties[2,3]. These difficulties are related to the comparable size between the drops and the pores in the surface of these materials. As a consequence, the drops can partially fit into the pores (or even fall completely into the pore if it is large enough) and the obtained measurement cannot be considered as an absolute contact angle (θ), but an apparent contact angle (γ) (see Figure S4).

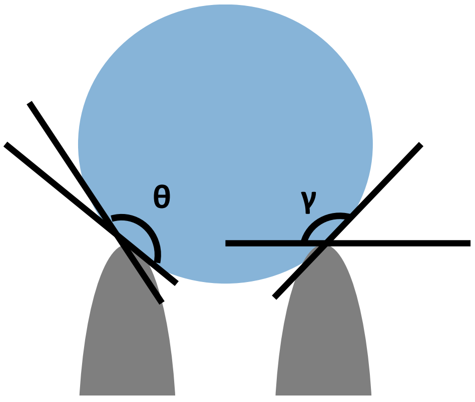

a)

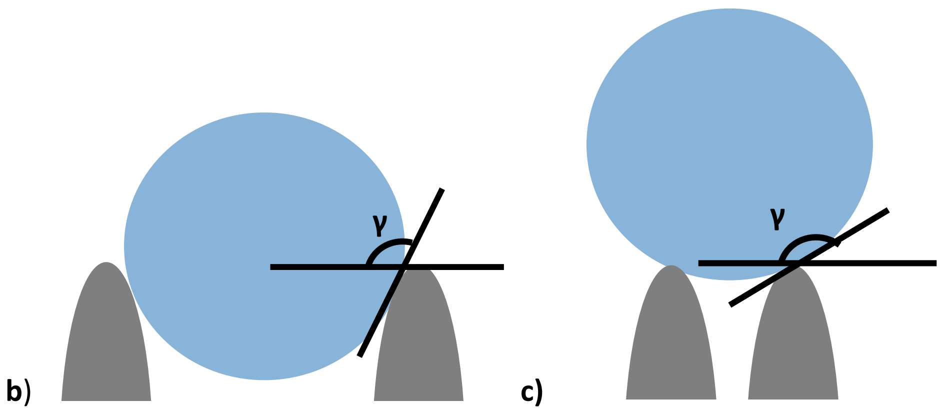

Figure S4. Water drop placed in a PU pore, where θ is the real contact angle of water (WCA), and γ is the apparent contact angle (APCA) measured by the optical contact angle goniometer (a). Figures (b) and (c) illustrate the influence of the pore size on the apparent contact angle of water drops with the same size.

This effect leads to a relationship between the pore size and the apparent contact angle for polymer foams with identical chemistry and surface roughness (in the polymer surface of the pore struts). Large pore sizes present lower AWCA, while smaller pore sizes present higher AWCA. As a direct relationship between this AWCA and the oil-water selectivity of the foams has been found, this apparent contact angle is considered a proper measurement for the characterization of porous materials in oil-water separation applications.[2]

Moreover, when comparing porous materials with the same pore size but different surface chemistry and/or roughness (in the polymer surface of the pore struts), the AWCA provides accurate information about the influence of the surface functionalization on the wetting properties of these materials.[1]

S3-Absorption tests.

Due to the buoyancy of the samples, and to increase the contact between the foams and the emulsions, the foams were forced and kept below the surface of the water during the absorption experiments (Figure S5).

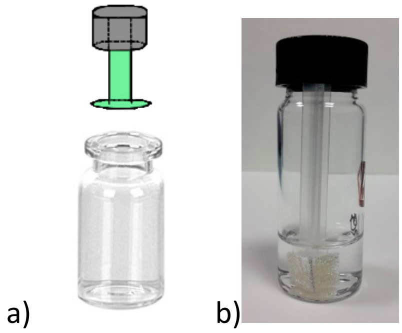

Figure S5. Schematic representation (a) and photograph (b) of the absorption tests set-up.

As explained in the manuscript, the functionalization procedure enhanced the oil absorption selectivity of the foams, showing negligible water uptakes but at the same time a slightly lower oil absorption capacity (Figure S6).

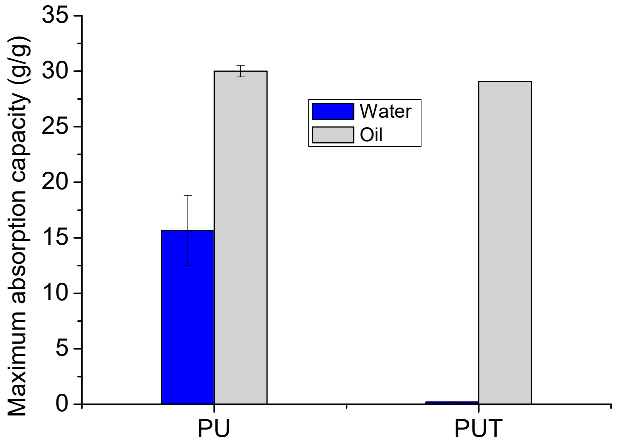

Figure S6. Water and oil absorption capacity using PU and PUT foams.

However, this slight decrease of the oil absorption capacity was expected due to the weight increase induced by the coating (i.e., the weight increase provided by the treatment affects the absorption capacity). Accordingly, it is possible to estimate theoretically the oil absorption capacity of the treated materials (C_{The}), taking into consideration the weight increase due to the treatment (wt.%) and the oil absorption capacity of the pristine materials (C_{oil}) using Equation 7[1].

$$C_{The} = \frac{C_{oil}}{(100 + wt.\%)} \cdot 100 \quad (7)$$

As shown in table S2, the estimation of the oil absorption capacity of the PUT foams accurately agreed with the experimental results.

Table S2. Oil and water absorption capacity of PU and PUT foams.

Foam	wt.%	C_{The} [g/g]	C_{oil} [g/g]	C_{water} [g/g]
PU	--	--	30.5 ± 0.5	15.64 ± 3.17
PUT (Spraying TPU / SNPs – 20 cm)	4.54	29.18	29.08 ± 0.02	0.20 ± 0.01

S4. FTIR

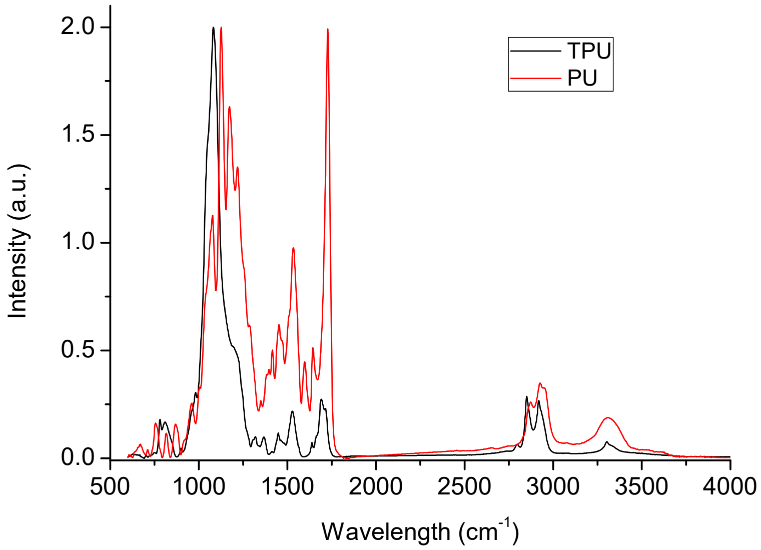
2.0
1.5
1.0
0.5
0.0
Intensity (a.u.)
500
1000
1500
2000
2500
3000
3500
4000
Wavelength (cm-1)
TPU
PU

a)

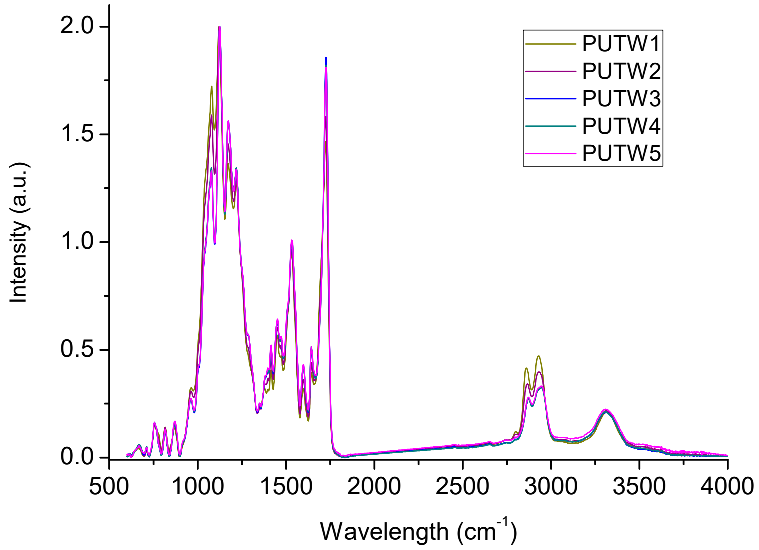
2.0
1.5
1.0
0.5
0.0
Intensity (a.u.)
500
1000
1500
2000
2500
3000
3500
4000
Wavelength (cm-1)
PUTW1
PUTW2
PUTW3
PUTW4
PUTW5

b)

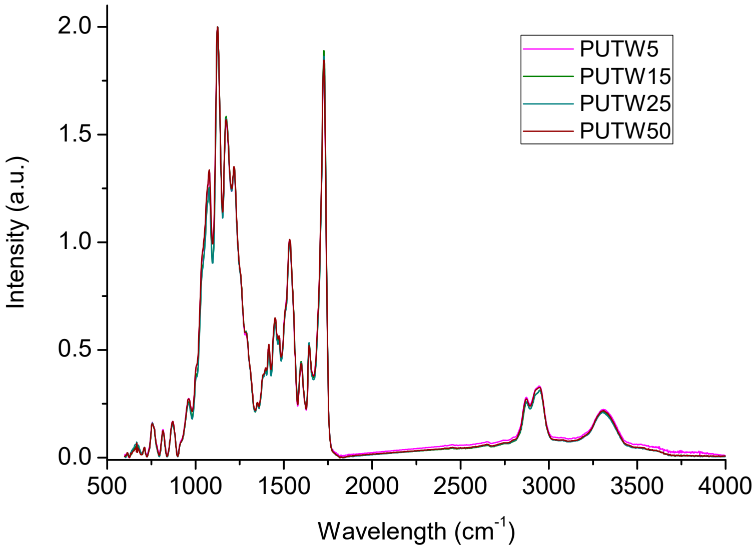

c)

Figure S7. FTIR spectra of PU foams and TPU (a), PUT samples after 1 (PUTW1), 2 (PUTW2), 3 (PUTW3), 4 (PUTW4), and 5 (PUTW5) washing cycles (b), and PUT samples after 5, 15 (PUTW15), 25 (PUTW20), and 50 (PUTW50) washing cycles (c).

S5. References

1. Pinto J.; Heredia-Guerrero J.A.; Athanassiou A.; Fragouli D. Reusable nanocomposite-coated polyurethane foams for the remediation of oil spills. *Int J Environ Sci Technol*. **2017**. doi:10.1007/s13762-017-1310-6.
2. Pinto J.; Athanassiou A.; Fragouli D. Effect of the porous structure of polymer foams on the remediation of oil spills. *J Phys D Appl Phys*. **2016**, *49*,145601–145608, doi:10.1088/0022-3727/49/14/145601.
3. Su C. Highly hydrophobic and oleophilic foam for selective absorption. *Appl Surf Sci*. **2009**, 256, 1413–1418. doi:10.1016/j.apsusc.2009.08.098.
